# Impact of Stress Hyperglycemia on Early Neurological Deterioration in Acute Ischemic Stroke Patients Treated With Intravenous Thrombolysis

**DOI:** 10.3389/fneur.2022.870872

**Published:** 2022-05-13

**Authors:** Ling Wang, Qiantao Cheng, Ting Hu, Nuo Wang, Xiu'e Wei, Tao Wu, Xiaoying Bi

**Affiliations:** ^1^Department of Neurology, Changhai Hospital, Second Military Medical University, Shanghai, China; ^2^Department of Neurology, The Fourth Affiliated Hospital of Anhui Medical University, Hefei, China; ^3^Department of Neurology, Affiliated Drum Tower Hospital of Nanjing University, Medical School, Nanjing, China; ^4^Department of Neurology, The Second Affiliated Hospital of Xuzhou Medical University, Xuzhou, China; ^5^Department of Neurology, Center of Cerebrovascular Disorders, Second Military Medical University, Shanghai, China

**Keywords:** acute ischemic stroke, stress hyperglycemia, early neurological deterioration, intravenous recombinant tissue-type plasminogen activator, stress hyperglycemia ratio

## Abstract

**Background and Purpose:**

It has been widely reported that stress hyperglycemia contributes to poor prognosis in patients experiencing acute ischemic stroke (AIS). However, its predictive value for early neurological deterioration (END) after intravenous administration of recombinant tissue-type plasminogen activator (IV-rtPA) in AIS patients is still unclear. The aim of this study was to evaluate the impact of stress hyperglycemia on the risk of END after IV-rtPA.

**Methods:**

A total of 798 consecutive patients treated with IV-rtPA were included in this study. The stress hyperglycemia ratio (SHR) was calculated as fasting plasma glucose level at admission (mg/dl)/glycosylated hemoglobin (HbAlc) (%). END was defined as a National Institutes of Health Stroke Scale Score (NIHSS) ≥ 4 points 24 h after IV-rtPA, and poor functional outcome at discharge was defined as a modified Rankin Scale (mRS) score of 3–6 at discharge. Patients with a prior history of diabetes or HbAlc ≥ 6.5% were considered to have diabetes mellitus. Patients were grouped according to SHR values. Multivariate logistical regression was used to evaluate the risk of END for patients within specific SHR categories.

**Results:**

In total, 139 (17.4%) patients had END. After adjusting for confounders, the highest tertile group had higher risks of END and poor functional outcome at discharge than those of patients in the lowest tertile group (*OR*, 1.95; 95% *CI*, 1.21–3.15; *p* = 0.006) (*OR*, 1.85; 95% *CI*, 1.163–2.941; *p* = 0.009), and the predictive value of high SHR for END was also significant in patients with diabetes mellitus (*OR*, 3.05; 95% *CI*, 1.29–7.21; *p* = 0.011). However, a significant association of high SHR and poor functional outcome was only found in patients without diabetes (*OR*, 1.85; 95% *CI*, 1.002–3.399; *p* = 0.045).

**Conclusion:**

A higher SHR predicted that patients with severe stress hyperglycemia had higher risks of END and poor functional outcome at discharge after IV-rtPA.

## Introduction

Early neurological deterioration (END) following acute ischemic stroke (AIS) in patients treated with intravenous recombinant tissue-type plasminogen activator (IV-rtPA) is a common and prominent clinical issue that is strongly associated with patient dependency and death at 3 months after AIS (1, 2). The incidence of END after IV-rtPA is ~8.1–28.1% (3). Cases of END can be divided into two categories based on whether or not there are clear causes. The common causes are symptomatic intracerebral hemorrhage (sICH), malignant edema, early recurrent stroke and early seizures (4, 5), and the remaining proportion of END cases that remain without a clear mechanism are called unexplained END. In recent years, several clinical trials have found that multiple factors, and particularly hemodynamic and metabolic factors, are associated with END (6). Hyperglycemia might be one of the factors that facilitates END (1, 7), but the conclusions of relevant studies on this association are still inconsistent (3).

Hyperglycemia is a common phenomenon that is present in half of patients with AIS (8), and the common triggers are poor control of chronic hyperglycemia, physiological stress, or both. When a serious illness occurs, both diabetic and non-diabetic patients may experience stress hyperglycemia. The degree of stress hyperglycemia may reflect the degree of physiological stress and the risk of poor prognosis. Stress-induced hyperglycemia might be a better marker of poor prognosis in many severe diseases than absolute hyperglycemia (9, 10). The impact of stress hyperglycemia on neurologic deficit and mortality after AIS has been confirmed previously (8, 11). In patients with AIS treated with IV-rtPA, severe stress hyperglycemia was significantly associated with symptomatic intracranial hemorrhage, poor outcome, and mortality after 3 months (12), but the adverse effect of stress hyperglycemia was more pronounced in non-diabetic patients (8). However, the association of stress hyperglycemia with END is undefined, thus, the aim of this study was to validate its predictive value for END in patients with AIS treated with IV-rtPA, and we further explored whether the predictive effect is different in diabetic and non-diabetic patients.

## Methods

### Study Population

In this retrospective study, data were collected from consecutive patients with AIS treated with IV-rtPA at the Department of Neurology, ChangHai Hospital and The Second Affiliated Hospital of Xu Zhou Medical School from January, 2017 to December, 2020. The inclusion criteria were as follows: (1) age of 18 years or older; (2) admission within 4.5 h after onset; and (3) treatment with IV-rtPA. The exclusion criteria were as follows: (1) diagnoses of malignant tumors, autoimmune diseases, major organ failure or presence of an active infection; and (2) incomplete clinical data. We excluded patients who received IV-rtPA and endovascular thrombectomy to reduce the heterogeneity of enrolled patients. Informed consent was obtained from participants or legal representatives, and the protocol was approved by the Ethics Committee of Changhai Hospital and the Second Affiliated Hospital of Xu Zhou Medical School.

### Data Collection

Patient demographics, vascular risk factors (such as hypertension, atrial fibrillation, coronary heart disease, history of stroke, smoking status, and drinking status), time from symptom onset to IV-rtPA treatment (OTT), and previous antiplatelet and laboratory data were collected from medical records. Diabetes was diagnosed based on a prior history of diabetes or an HbA1c level of ≥6.5% (13). The severity of stroke was assessed with the National Institutes of Health Stroke Scale (NIHSS), and stroke subtype was classified according to the Trial of Org 10,172 in Acute Stroke Treatment (TOAST) criteria (14). The neutrophil to lymphocyte ratio (NLR) was calculated as neutrophil counts/lymphocyte counts. The diagnosis of sICH was based on the results of CT scans and defined according to the Heidelberg Bleeding Classification (15). Early malignant edema was considered, if the brain was swollen, a midline shift was present, and consciousness worsened. A poor functional outcome at discharge was defined as a modified Rankin Scale (mRS) score of 3–6 at discharge.

### Definition of END

In this study, END was defined as a ≥4-point increase in NIHSS score between the time of admission and 24 h after IV-rtPA (3). Neurological deficit was evaluated on admission and at 24 h after IV-rtPA by two certified neurologists blinded to the clinical data.

### Assessment of Stress Hyperglycemia

Fasting plasma glucose levels were monitored shortly after admission before IV-rtPA. Glycosylated hemoglobin (HbAlc %) was measured within 24 h after hospitalization, and stress hyperglycemia was calculated with the stress hyperglycemia ratio (SHR) as fasting plasma glucose levels (mg/dl)/HbA1c (%). Patients were stratified into three groups (T1–T3) based on the SHR value; the higher the SHR index, the more severe the stress hyperglycemia was considered.

### Statistical Analysis

The Kolmogorov–Smirnov test was performed to test the normality of variables, and continuous variables were described as the mean (standard deviation) and median (quartile) based on the normality of the data. Categorical variables are expressed as percentages. Differences in the baseline characteristics were assessed by χ^2^-test for categorical variables and ANOVA or Kruskal–Wallis test for continuous variables.

Multivariable binary logistical regression was used to analyze the predictive value of the SHR for END and poor functional outcome at discharge for all patients as well as patients grouped based on diabetes diagnosis. We used the lowest SHR tertile as the reference category. The covariates entered in the multivariable logistical regression were age, admission NIHSS, OTT, proximal artery occlusion, stroke subtype, sICH, malignant edema, and NLR. The two-tailed *p-*values of <0.05 were considered statistically significant. Data analyses were performed using the statistical software package SPSS 22.0 for Windows (IBM, Armonk, NY).

## Results

### Baseline Characteristics

During the study period, we retrospectively included 892 patients with AIS treated with IV-rtPA. Of those, 69 patients had no data on HbA1c levels, 20 patients had no data on baseline NIHSS score at admission, and eight patients had no data on admission blood glucose levels. After excluding the above patients, a total of 798 eligible patients were included in this study: 139 (17.4%) patients had END, 215 (26.9%) patients had poor functional outcomes at discharge, and 10 (1.3%) patients died during hospitalization.

Baseline characteristics are presented in Table 1. Patients in the END group were more likely to be older (69 vs. 66, *p* = 0.008), to present with a higher admission NIHSS score (8 vs. 4, *p* < 0.001), to have proximal artery occlusion (13.7 vs. 4.6%, *p* < 0.001), to have a higher incidence of large artery atherosclerosis (41.6 vs. 29.6%, *p* = 0.001), to have lower platelet counts (204.0 vs. 216.3, *p* = 0.038), and to have a higher NLR (2.7 vs. 2.5, *p* = 0.026) and SHR (1.4 vs. 1.2, *p* < 0.001).

**Table 1:** Baseline characteristics of subgroups based on the presence of post-thrombolysis early neurological deterioration.

	**Total**	**END group**	**Non-END group**	***p***
	**(*n* = 798)**	**(*n* = 139)**	**(*n* = 659)**	
**Demographics**				
Age, mean (SD), y	67 (11.4)	69 (12.5)	66 (11.1)	0.008
Male, *n* (%)	512 (64.2)	93 (66.9)	419 (63.6)	0.260
**Vascular risk factors**				
Hypertension, *n* (%)	543 (68.3)	99 (71.7)	444 (67.6)	0.197
Diabetes mellitus, *n* (%)	227 (28.6)	45 (32.6)	182 (27.7)	0.255
Atrial fibrillation, *n* (%)	110 (13.9)	22 (15.9)	88 (13.5)	0.498
Coronary heart disease, *n* (%)	101 (12.8)	16 (11.6)	85 (13.0)	0.779
Previous stroke, *n* (%)	155 (19.5)	27 (19.6)	128 (19.5)	0.532
Current smoking, *n* (%)	319 (40.2)	57 (41.3)	262 (40.0)	0.424
Current drinking, *n* (%)	187 (23.6)	35 (25.4)	152 (23.2)	0.326
**Clinical data**
SBP, mean (SD), mmHg	153 (21.5)	155 (21.1)	153 (21.6)	0.192
DBP, mean (SD), mmHg	88 (13.8)	89 (13.0)	87 (14.9)	0.170
Admission NIHSS, median (IQR)	5 (3–10)	8 (4–12)	4 (3–9)	<0.001
Previous antiplatelet, *n* (%)	149 (18.7)	124 (18.8)	25 (18.0)	0.905
Proximal artery occlusion, *n* (%)	49 (6.1)	19 (13.7)	30 (4.6)	<0.001
OTT, mean (SD), min	147 (59.5)	145 (59.0)	148 (60)	0.659
Stroke subtype, *n* (%)				0.001
LAA	233 (29.5)	57 (41.6)	176 (29.6)	
CE	105 (13.3)	22 (16.1)	83 (12.7)	
SAO	347 (43.7)	44 (32.1)	303 (46.3)	
Others and undetermined	106 (13.4)	14 (10.2)	92 (14.1)	
**Laboratory data**				
PLT, mean (SD)	214.1 (63.1)	204.0 (57.7)	216.3 (64.0)	0.038
TG, mean (SD), mmol/L	1.6 (1.2)	1.5 (1.0)	1.6 (1.2)	0.323
LDL	3.0 (1.0)	3.1 (0.9)	3.0 (1.0)	0.119
FIB, mean (SD), g/L	3.6 (0.9)	3.6 (0.8)	3.6 (0.9)	0.506
Homocysteine, mean (SD), umol/L	16.3 (14.8)	17.2 (19.4)	16.1 (13.6)	0.451
WBC, mean (SD)	8.0 (2.6)	8.3 (2.8)	7.9 (2.6)	0.055
NLR	2.5 (1.7–4)	2.7 (1.9–5.2)	2.5 (1.6–3.9)	0.026
SHR	1.3 (0.4)	1.4 (0.3)	1.2 (0.4)	<0.001

*SD, standard deviation; IQR, interquartile range; SBP, Systolic Blood Pressure; DBP, Diastolic Blood Pressure; NIHSS, National Institutes of Health Stroke Scale; OTT, Onset-to-treatment; LAA, Large Artery Atherosclerosis; CE, Cardioembolism; SAO, Small Artery Occlusion; PLT, Platelet; TG, Triglyceride; LDL, Low-density Lipoprotein; FIB, Fibrinogen; PLT, Platelet; WBC, White blood cell counts; NLR, Neutrophil-to-lymphocyte ratio; SHR, stress hyperglycemia ratio*.

The baseline characteristics of patients grouped according to the SHR value are provided in Table 2. Patients in the higher SHR group were more likely to have diabetes mellitus (29.3 vs. 25.2% vs. 52.3%, *p* < 0.001); to have a higher admission NIHSS score (6 vs. 7 vs. 8, *p* = 0.003); to have a higher incidence of large artery atherosclerosis (24.2 vs. 27.4 vs. 36.8%, *p* = 0.048); to have a higher white blood cell count (7.6 vs. 8.2 vs. 8.1, *p* = 0.012), and NLR (2.3 vs. 2.5 vs. 2.8, *p* < 0.001); and to have a higher incidence of END (12.8 vs. 14.7 vs. 24.8%, *p* < 0.001), and poor functional outcome at discharge (19.9 vs. 24.1 vs. 36.8, *p* < 0.001).

**Table 2:** Baseline characteristics of subgroups based on SHR ratio.

	**T1 (266)**	**T2 (266)**	**T3 (266)**	***p***
**Demographics**				
Age, mean (*SD*), y	66 (11.2)	67 (11.2)	67 (11.8)	0.712
Male, *n* (%)	186 (69.9)	161 (60.5)	165 (62.0)	0.520
**Vascular risk factors**				
Hypertension, *n* (%)	171 (64.3)	193 (72.6)	179 (67.3)	0.117
Diabetes mellitus, *n* (%)	78 (29.3)	67 (25.2)	139 (52.3)	<0.001
Atrial fibrillation, *n* (%)	30 (11.3)	45 (16.9)	35 (13.2)	0.158
Coronary heart disease, *n* (%)	30 (11.3)	34 (12.8)	37 (13.9)	0.657
Previous stroke, *n* (%)	55 (20.7)	50 (18.8)	50 (18.8)	0.819
Current smoking, *n* (%)	123 (46.2)	104 (39.1)	92 (34.6)	0.022
Current drinking, *n* (%)	63 (23.7)	63 (23.7)	61 (22.9)	0.978
**Clinical data**
SBP, mean (SD), mmHg	151 (18.8)	155 (22.7)	153 (22.8)	0.081
DBP, mean (SD), mmHg	87 (13.2)	89 (14.8)	87 (13.1)	0.131
Admission NIHSS, mean (SD)	6 (5.5)	7 (6.2)	8 (6.4)	0.003
Previous antiplatelet, *n* (%)	54 (20.3)	47 (17.7)	48 (18.0)	0.701
Malignant edema, *n* (%)	2 (0.8)	2 (0.8)	4 (1.5)	0.603
sICH, *n* (%)	2 (0.8)	7 (2.6)	6 (2.3)	0.240
END, *n* (%)	34 (12.8)	39 (14.7)	66 (24.8)	<0.001
Proximal artery occlusion, *n* (%)	13 (4.9)	16 (6.0)	20 (7.5)	0.447
OTT, median (IQR), min	142 (98.7–200.0)	151 (93.0–199.0)	147 (103.0–180.5)	0.850
Stroke subtype, *n* (%)				0.048
LAA	65 (24.2)	73 (27.4)	98 (36.8)	
CE	33 (12.4)	39 (14.7)	33 (12.4)	
SAO	126 (47.4)	122 (45.9)	103 (38.7)	
Others and undetermined	42 (15.8)	32 (12.0)	32 (12.0)	
Poor outcome at discharge, *n* (%)	53 (19.9)	64 (24.1)	98 (36.8)	<0.001
In hospital mortality, *n* (%)	2 (0.8)	4 (1.5)	4 (1.5)	0.667
**Laboratory data**				
FBG	5.7 (5.2–6.2)	6.9 (6.5–7.7)	10.6 (8.5–15.1)	<0.001
PLT, mean (SD)	214.6 (63.9)	216.9 (64.1)	211.0 (61.4)	0.555
TG, mean (SD), mmol/L	1.6 (1.0)	1.6 (1.4)	1.6 (1.0)	0.861
LDL, mean (SD), mmol/L	3.0 (0.9)	3.1 (1.0)	3.0 (0.9)	0.605
FIB, mean (SD), g/L	3.6 (0.8)	3.6 (0.8)	3.6 (1.0)	0.833
Homocysteine, mean (SD), umol/L	16.3 (13.1)	16.3 (14.6)	16.3 (14.8)	0.999
WBC, mean (SD)	7.6 (2.4)	8.2 (2.7)	8.1 (2.6)	0.012
NLR, median (IQR)	2.3 (1.6-3.5)	2.5 (1.6-4.1)	2.8 (1.9-4.5)	<0.001

*SD, standard deviation; IQR, interquartile range; SBP, Systolic Blood Pressure; DBP, Diastolic Blood Pressure; NIHSS, National Institutes of Health Stroke Scale; sICH, symptomatic intracranial hemorrhage; OTT, Onset-to-treatment; LAA, Large Artery Atherosclerosis; CE, Cardioembolism SAO, Small Artery Occlusion; PLT, Platelet; TG, Triglyceride; LDL, Low-density Lipoprotein; FIB, Fibrinogen; PLT, Platelet; WBC, White blood cell counts; NLR, Neutrophil-to-lymphocyte ratio*.

### Association of the SHR With END and Poor Functional Outcome at Discharge in Multivariate Analysis

Multivariate logistical regression was performed to investigate the association of the SHR with END and poor functional outcome at discharge for all patients and for the subgroups of patients with or without diabetes. The results are displayed in Tables 3, 4. After adjusting for confounders, the risks of END and poor functional outcome at discharge in the third tertile group was significantly higher than those of patients in the first tertile group (as the reference value; *OR*, 1.95; 95% *CI*, 1.21–3.15; *p* = 0.006; *OR*, 1.85; 95% *CI*, 1.163–2.941; *p* = 0.009). Patients were further divided into two groups according to whether they had diabetes, and the predictive effect of severe stress hyperglycemia on END was significant only for diabetic patients (*OR*, 3.05; 95% *CI*, 1.29–7.21; *p* = 0.011), and showed a trend for predicting END in non-diabetic patients (*OR*, 1.32; 95% *CI*, 0.694–2.509; *p* = 0.398). Conversely, the relationship of severe hyperglycemia with poor functional outcome at discharge (*OR*, 1.85; 95% *CI*, 1.002–3.399; *p* = 0.045) was significant only in patients without diabetes.

**Table 3:** Multivariate logistical regression analyses depicting the association of SHR and respective outcomes.

**Outcome**	**Variable**	***OR***	**95% *CI***	***p***
END	SHR distribution			
	T1	Reference	Reference	
	T2	0.92	0.541–1.579	0.772
	T3	1.95	1.207–3.149	0.006
	SHR distribution			
Poor outcome at	T1	Reference		
discharge	T2	0.97	0.591–1.593	0.906
	T3	1.85	1.163–2.941	0.009

*END, Early Neurological Deterioration; CI, Confident Interval; OR, Odds Ratio; SHR, stress hyperglycemia ratio; P for trend*.

**Table 4:** Multivariate logistical regression analyses depicting the association of SHR and END in the patients with and without diabetes.

**Patients**	**SHR distribution**	***OR***	***CI***	***p***
**END**				
Without diabetes	T1	Reference		
	T2	0.75	0.401–1.419	0.382
	T3	1.32	0.694–2.509	0.398
With diabetes	T1	Reference		
	T2	1.81	0.663–4.954	0.246
	T3	3.05	1.288–7.210	0.011
**Poor outcome at discharge**				
Without diabetes	T1	Reference		
	T2	0.90	0.496–1.629	0.725
	T3	1.85	1.002–3.399	0.049
With diabetes	T1	Reference		
	T2	1.44	0.569–3.661	0.439
	T3	1.79	0.808–3.965	0.151

*END, Early Neurological Deterioration; OR, Odds ratio; CI, Confident Interval; SHR, stress hyperglycemia ratio; P for trend*.

## Discussion

This study indicated that the SHR was independently associated with increased risks for END and poor functional outcome at discharge in AIS patients treated with IV-rtPA. Furthermore, the association of the SHR with END in the subgroup of patients who were diagnosed with diabetes mellitus was also observed. The predictive value of SHR values for poor functional outcome at discharge is more apparent in patients without diabetes.

Recently, the impact of hyperglycemia on END in patients with AIS has been considered controversial, and it might be a contributing factor rather than a direct cause (3). Previous studies used absolute hyperglycemia as an observational index while ignoring differences in patient baseline glycemia, and this practice may affect analyses of the relationship of hyperglycemia with disease prognosis. Therefore, stress hyperglycemia was introduced into the analysis to control for baseline glycemia and to reflect the relative increase in glycemia. The occurrence of stress hyperglycemia involves activation of the hypothalamic-pituitary axis and sympatho-adrenal system and increases the release of epinephrine, norepinephrine, and pro-inflammatory cytokines (TNF-α, IL-1, and IL-6) (16). The underlying mechanism of the effect of stress hyperglycemia on END may involve the following aspects. First, stress hyperglycemia is associated with greater inflammatory and neurohormonal responses, increased induction of endothelial apoptosis, and increased oxidative stress (17). Oxidative stress activates matrix metalloproteinase gelatinase B (MMP-9), mediates the breakdown of the blood–brain barrier (BBB), aggregates BBB leakage, and increases the risk of brain edema, and hemorrhage after reperfusion (18). Previous studies have confirmed that stress hyperglycemia has a certain predictive value for hemorrhagic transformation in patients with AIS (19). Second, stress hormones can stimulate hepatic gluconeogenesis and inhibit glucose uptake in peripheral tissues (16). Pro-inflammatory cytokines may contribute to the upregulated expression and membrane localization of glucose transporters GLUT-1 and GLUT-3 and the facilitation of glucose uptake, and they are utilized by insulin-independent tissues such as the peripheral and central nervous system (20). Cellular glucose overload increases brain lactate production and further promotes the conversion of asymptomatic tissue into symptomatic tissue (21). Third, both acute and chronic hyperglycemia play an important role in prothrombotic shift (22) and may facilitate thrombus extension, which has a strong association with END (3). Many factors are involved in this process, such as hyperglycemia and hyperinsulinemia, which can increase the expression of plasminogen activator inhibitors-1 (PAI-1); as a result, the activity of rt-PA is reduced (23). Hyperglycemia can disrupt the endothelial glycocalyx layer, induce the release of coagulation factors harbored in the layer, and enhance platelet-endothelial adhesion (24). Many complex pathways seem to be involved in the process of hyperglycemia-induced hypercoagulability, and more research is needed for further clarification. Forth, stress hyperglycemia may reflect transient glycemic variability. The glucose fluctuations exhibited a more specific triggering effect on oxidative stress (25). Previous study have shown the greater glycemic variability of patient with AIS may be correlated with lower odds of neurological improvement during hospitalization (26), and the link between glycemic variability and END may also exist due to the increased oxidative stress. Finally, the degree of stress hyperglycemia may reflect the severity of diseases; in patients with AIS, it can represent the extent of ischemic damage, and severe neurological impairment was significantly associated with END (3).

Consistent with the results of previous studies (8), we found that severe stress hyperglycemia also predicts poor functional outcome at discharge. First, hyperglycemia may be directly toxic to the ischemic brain, especially the ischemic penumbra, and lactate accumulation, intracellular acidosis, enhanced lipid peroxidation, and free radical formation may all contribute to this process (27). Thus, hyperglycemia may promote damage to potentially salvageable neurons in the ischemic penumbra of the infarction (28). Second, END is an independent predictor of poor prognosis, and the possible mechanisms by which stress hyperglycemia affects END mentioned above may also explain this finding.

In this study, we found that in the diabetes mellitus group, patients with stress hyperglycemia were more prone to END after treatment with IV-rtPA. This conclusion is consistent with those of previous studies reporting that higher glycemic variability in diabetic patients significantly increased the risk of post-stroke cardiovascular mortality and END (29, 30). Ostensibly, when assessed relative to baseline glucose levels, stress hyperglycemia is a kind of hyperglycemia that can more comprehensively reflect the instability of blood glucose levels. The underlying mechanism is not fully understood. A possible explanation is that chronic hyperglycemia can cause microvascular remodeling, increase the thickness of the capillary basement membrane, alter capillary density, and influence collateral circulation (31). Patients with diabetic microangiopathy were more sensitive to ischemia and hypoxia, and the cellular glucose overload induced by stress hyperglycemia increases the consumption of intracellular oxygen and thereby results in a deepening and an extension of hypoperfusion. Several studies have confirmed that diabetes mellitus abolishes the protective effects of ischemic preconditioning. A transient ischemia–reperfusion process may contribute to tissue protection, and diabetes can increase oxidative stress and levels of reactive oxygen species and inhibit autophagy to attenuate remote preconditioning-induced tissue protection (32). In the subgroup analysis, the impact of severe stress hyperglycemia on poor prognosis was found to be more pronounced in non-diabetic patients. Some previous studies had similar findings (8, 11), in non-diabetic patients, stress hyperglycemia is associated with increased risk of neurological deficit, mortality, and stroke recurrence. Even, we found that patients with diabetes developed stress hyperglycemia are more likely to experience END, the possible hypotheses are that diabetic patients with chronic exposure to high concentrations of blood glucose may develop tolerance mechanisms that offset adverse metabolic effects, and the glycemic threshold in chronic hyperglycemia patients with severe stress hyperglycemia is higher than that in normoglycemic patients, indicating a stronger physical stress response. Furthermore, the deleterious effects of stress hyperglycemia may have acute and long-term outcome depending on the preischemic glucose metabolism. This may explain that in this study, diabetic patients with stress hyperglycemia were more prone to END, but did not increase the risk of poor prognosis.

In China, the Ministry of Health China Stroke Prevention Project Committees (CSPPC) was established to monitor the quality of stroke care in stroke center hospitals (33). The CSPPC actively promotes the construction of stroke center network, stroke map and stroke “Green Channel,” effectively shorten the prehospital time and improved the prognosis of patients with stroke (33, 34). However, this study suggests that even in patients eligible for thrombolysis, there are still some inherent risk factors affecting END and prognosis. Therefore, it is crucial to find biomarkers that are easily available clinically and have reliable predictive effects. As a novel index to quantify stress hyperglycemia, this study highlights the importance of intervention and monitoring glycemia and suggests that the assessment of glucose should be combined with information on the patient's baseline glycemia. But there is no universally accepted insulin interventions, a multicenter randomized clinical trial suggested that intensive glucose control in patients with AIS with hyperglycemia did not significant improve functional outcome (35). This conclusion may be attributed to the higher incidence of hypoglycemia in the intensive treatment group. Regarding whether SHR can instead of absolute hyperglycemia to become a treatment target, further investigations is warranted.

This study has several limitations that should be addressed when interpreting the results. First, this is a retrospective study; ~1-10th of the patients were excluded because they lacked clinical data, and we excluded patients treated with endovascular therapy after IV-rtPA, which inevitably produced biases. Second, this study used admission blood glucose to calculate SHR, and other time points of glycemic changes and control status were not considered. Third, we diagnosed diabetes based on past-medical history and HbA1c and did not further determine the patient's previous glycemic status. Fourth, this study was performed in a single country, which might limit the generalizability of the results to other patient cohorts. However, the strengths of this study include the large sample size and the fact that patients were selected from multiple centers *via* strict inclusion and exclusion criteria.

## Conclusion

In conclusion, stress hyperglycemia assessed by SHRs can be a useful predictive marker of END and poor prognosis at discharge after IV-rtPA, and this predictive value is related to diabetes status.

## Data Availability Statement

The raw data supporting the conclusions of this article will be made available by the authors, without undue reservation.

## Ethics Statement

The studies involving human participants were reviewed and approved by Changhai Hospital Ethics Committee and the Second Affiliated Hospital of Xu Zhou Medical School Ethics Committee. The patients/participants provided their written informed consent to participate in this study.

## Author Contributions

LW, QC, and XB conceived and designed the study. LW wrote the manuscript and performed the statistical analysis. LW, TH, and NW collected data and organised the database. LW, TH, QC, and XW reviewed and edited the manuscript, QC, XW, and XB contributed resources. TW contributed to data curation and supervision. All authors read and approved the manuscript.

## Conflict of Interest

The authors declare that the research was conducted in the absence of any commercial or financial relationships that could be construed as a potential conflict of interest.

## Publisher's Note

All claims expressed in this article are solely those of the authors and do not necessarily represent those of their affiliated organizations, or those of the publisher, the editors and the reviewers. Any product that may be evaluated in this article, or claim that may be made by its manufacturer, is not guaranteed or endorsed by the publisher.
